# Altered Brain Functional Asymmetry in Patients With Major Depressive Disorder Related to Gastrointestinal Symptoms

**DOI:** 10.3389/fnins.2021.797598

**Published:** 2022-02-17

**Authors:** Xiaoya Fu, Yudan Ding, Jindong Chen, Feng Liu, Huabing Li, Jingping Zhao, Wenbin Guo

**Affiliations:** ^1^National Clinical Research Center for Mental Disorders, Department of Psychiatry, The Second Xiangya Hospital of Central South University, Changsha, China; ^2^Department of Radiology, Tianjin Medical University General Hospital, Tianjin, China; ^3^Department of Radiology, The Second Xiangya Hospital of Central South University, Changsha, China; ^4^Department of Psychiatry, The Third People’s Hospital of Foshan, Foshan, China

**Keywords:** parameter of asymmetry (PAS), major depressive disorder (MDD), gastrointestinal (GI) symptoms, asymmetry, interhemispheric connectivity

## Abstract

**Objective:**

Disrupted brain functional asymmetry has been reported in major depressive disorder (MDD). The comorbidity may be a crucial factor to this functional asymmetry. It is quite common that gastrointestinal (GI) symptoms are comorbid with MDD, but limited evidence focuses on the effect of GI comorbidity on the neuropathology of MDD from a functional lateralization perspective.

**Methods:**

Resting-state functional magnetic resonance imaging was obtained in 28 healthy controls (HCs), 35 MDD patients with GI symptoms (GI-MDD patients), and 17 patients with MDD without GI symptoms (nGI-MDD patients). The parameter of asymmetry (PAS) was used to analyze the imaging data and evaluate the changes of functional asymmetry.

**Results:**

The GI-MDD patients showed increased PAS scores in the left inferior frontal gyrus (IFG) and superior medial prefrontal cortex (MPFC) and decreased PAS scores in the right postcentral gyrus in comparison with nGI-MDD patients. The PAS scores of the left IFG and left superior MPFC were correlated with the severity of GI problems and could be applied to distinguish GI-MDD patients from nGI-MDD patients with an accuracy, a sensitivity, and a specificity of 92.31, 100, and 76.47%, respectively. Furthermore, GI-MDD and nGI-MDD patients both displayed increased PAS scores in the PCC/precuneus.

**Conclusions:**

This study revealed the influence of concomitant GI symptoms on functional asymmetry in MDD patients. Increased PAS scores of the left IFG and superior MPFC might represent an unbalanced regulation of brain over GI function and had the potential to be regarded as distinctive features related to functional GI symptoms in MDD.

## Introduction

Hemispheric asymmetry is widespread in various species and of great significance for human perception, emotion, cognition, and behavior (Güntürkün et al., 2020). The hemispheric specialization is advantageous for individuals to efficiently process information and reduce reaction time. Generally, the left hemisphere appears to be more specialized for language and motor coordination, while the right hemisphere is more relevant to memory and visuospatial attention (Gotts et al., 2013; Caeyenberghs and Leemans, 2014).

The right-left asymmetry in the process of emotional information sparks an interest to researchers. Many different hypotheses of brain lateralization in the emotional processing were proposed, like the right hemisphere model (the dominance of the right hemisphere) and the approach-avoidance model (the opposite dominance of the left hemisphere for approach/positive affect and the right for avoidance/negative affect) (Gainotti, 2019). But the results of neuroimaging studies suggested that brain functional lateralization in the emotional process might be region-specific (Wager et al., 2003; Beraha et al., 2012). Though the pattern of lateralization in the emotional processing is still controversial, disrupted functional lateralization has been reported in depression, a disease with abnormal emotional processing. A meta-analysis of electroencephalograph (EEG) studies reported a link of depression to altered resting frontal asymmetry with a moderate effect (Thibodeau et al., 2006). The psychosocial risk, especially maternal depression, showed an association to greater right-sided resting frontal EEG asymmetry in children (Peltola et al., 2014). These findings indicate that the anomalous hemispheric asymmetry may be of great importance for clinical diagnosis and treatment of depression. Bruder et al. (2017) reviewed electrophysiological and functional magnetic resonance imaging (fMRI) evidence of brain asymmetry in depression and revealed abnormalities in brain asymmetry and lateralized responses to emotional stimuli, but the comorbidity might be a confound factor to suppress or enhance the alteration of brain asymmetry.

It is not rare that patients with major depressive disorder (MDD) are comorbid with other mental or somatic symptoms. Gastrointestinal (GI) symptoms and decreased appetite are fairly common manifestations in MDD patients. Over 70% of patients suffered from concomitant GI symptoms in depressive episodes (Huang J. et al., 2021). The presence of GI symptoms would have a negative influence on the course of MDD, contributing to greater depressive severity (Huang M. H. et al., 2021). Previous studies have reported that the concomitant GI symptoms in MDD were related to abnormal brain functional activity (Liu et al., 2020; Yan et al., 2021). Some work in functional GI disorders also found abnormal functional connectivity in the patients (Li et al., 2021). But limited studies focused on the effect of GI comorbidity on the neuropathology of MDD from a perspective of functional lateralization. Thus, we intended to investigate the abnormality of brain functional asymmetry in MDD patients with GI discomfort for a better understanding of the neuropathological influence of comorbid with GI symptoms on MDD.

We employed parameter of asymmetry (PAS) to reflect the change of functional asymmetry. PAS is a novel voxel-wise quantitative index defined as the difference of functional connectivity (FC) of the given voxel between voxels in contralateral and ipsilateral hemisphere. According to a previous study, both intra- and inter-hemispheric connectivity should be taken into consideration when assessing hemispheric specialization (Mueller et al., 2015). The communication and coordination between two hemispheres are of great significance for efficient process of complex and complementary cognitive tasks (Hoptman and Davidson, 1994; Güntürkün et al., 2020). PAS has been applied in exploring the anomalous asymmetry in many psychiatric disorders (Zhu et al., 2018, 2019; Su et al., 2020; Ding et al., 2021; Jia et al., 2021). In the present study, we investigated functional asymmetry in MDD patients with concomitant GI symptoms with this novel approach.

## Materials and Methods

### Participants

This study included 28 healthy controls (HCs) and 52 MDD patients following the DSM-5 criteria. Based on the existence of GI symptoms, 52 patients were classified as GI-MDD (MDD with at least one GI symptoms, *n* = 35) or nGI-MDD (MDD without GI symptoms, *n* = 17) patients. The GI symptoms mainly included medically unexplained nausea, vomit, constipation, diarrhea, gastralgia, heartburn, flatulence, and so on. All participants were Han Chinese and right-handed. Participants with organic digestive diseases, neurological disorders, severe physical diseases, brain structural abnormalities, pregnancy, and history of substance abuse or MRI scanning contraindications were excluded. HCs had no history of psychotic symptoms and were ruled out if they or their relatives had a history of mental disorders. All included patients scored 17 points or above in the 17-item Hamilton Rating Scale for Depression (HRSD-17) and had no history of antidepressants or electroconvulsive therapy. The severity of clinical symptoms of patients was assessed by HRSD-17 from the following five aspects: retardation symptoms (items 1, 7, 8, and 14), cognitive disturbances (items 2, 3, and 9), insomnia (items 4, 5, and 6), anxiety/somatization (items 10, 11, 12, 13, 15, and 17), and weight loss (item 16). The severity of GI symptoms was evaluated by GI symptoms item (item 12) in the HRSD-17.

This study was approved by the Medical Research Ethics Committee of the Second Xiangya Hospital of Central South University. All participants provided written informed consents.

### Image Acquisition and Data Preprocessing

Scanning was conducted on a Siemens 3.0T scanner, with headphones and foam padding to minimize head motion and scanner noises. Participants were instructed to remain motionless, close their eyes, and stay awake during scan. Echo planar imaging sequence was employed to obtain functional magnetic resonance imaging (fMRI) data with the following parameters: repetition time/echo time = 2,000 ms/30 ms, flip angle = 90°, field of view = 240 mm × 240 mm, matrix = 64 mm × 64, 4 mm slice thickness, 0.4 mm gap, 30 slices, number of volumes = 250.

The images were preprocessed by the Data Processing Assistant for Resting-State fMRI (DPARSF) software package (Yan and Zang, 2010). After removing the initial 10 volumes, slice-timing correction and head motion correction were conducted. Participants with excessive head movement (maximal translation > 2 mm or maximal rotation > 2°) would be excluded. The images were spatially normalized to a standard Montreal Neurological Institute template and resampled to 3 mm × 3 mm × 3 mm. A 4-mm Gaussian kernel of full-width at half-maximum was used in smoothing. Friston-24 head motion, signals of cerebrospinal fluid, and white matter were regressed out. After bandpass-filtered (0.01–0.08 Hz) and linearly detrended, the images were scrubbed (framewise displacement threshold of 0.2 mm).

### Parameter of Asymmetry Calculation

As described in previous studies (Zhu et al., 2018; Ding et al., 2021), the calculation of PAS scores followed the formula:


$$P\,A\,S=F\,C_{i\,n\,t\,e\,r}-F\,C_{i\,n\,t\,r\,a}$$

*FC*_*inter*_ and *FC*_*intra*_ refer to interhemispheric FC and intrahemispheric FC, respectively. *FC*_*inter*_ is defined as the mean FC (Fisher’s *Z*-transformed) of a given voxel between other voxels in the contralateral hemisphere, whereas *FC*_*intra*_ is the mean FC of the given voxel between the voxels in the ipsilateral hemisphere. Negative correlations were not included in calculation for their damaging effect on reliability (Wang et al., 2011). A threshold of *r* > 0.2 was set to remove weak correlations possibly resulting from signal noises (Wang et al., 2013).

### Statistical Analysis

Two-sample *t*-tests, one-way analyses of variance, and chi-square test were used to analyze demographic and clinical data based on the scale of measure. A threshold of *p* < 0.05 was set as the significant level.

PAS scores were compared by using analyses of covariance (ANCOVA) across three groups, followed by *post hoc t*-tests for multiple comparison, with age, gender, years of education, and the mean framewise displacement as covariates. A threshold of *p* < 0.05 was set as the significant level for the false discovery rate correction.

The PAS scores of clusters showed significant group differences were extracted for further correlation and support vector machine (SVM) analysis. Spearman correlation followed by the Benjamini–Hochberg correction was used in correlation analysis between the extracted PAS scores and clinical variables in the patients. SVM analysis was applied to examine whether the extracted PAS scores were capable of discriminating GI-MDD and nGI-MDD patients. The analysis employed a “leave-one-out” strategy using the LIBSVM software package (Chang and Lin, 2011).

## Results

### Demographic and Clinical Characteristics

No participant was excluded because of excessive head movement. As demographic and clinical details presented in Table 1, there was no apparent difference in age, sex ratio, and years of education across three group. Two patient groups had no significant differences in illness duration. Relative to nGI-MDD patients, GI-MDD patients experienced more severe depressive symptoms, indicated by higher HRSD-17 scores, particularly in the factors of anxiety/somatization, weight loss, and sleep disturbance.

**TABLE 1:** Demographic and clinical characteristics of the participants.

	GI-MDD (*n* = 35)	nGI-MDD (*n* = 17)	HCs (*n* = 28)	*F, t* or χ*^2^* value	*Post hoc t*-tests or *p*-values
Age (years)	30.86 ± 6.84	30.29 ± 8.05	30.14 ± 5.00	0.102	0.90^a^
Gender (male/female)	13/22	6/11	14/14	1.377	0.50^b^
Education (years)	14.51 ± 3.28	12.94 ± 3.46	14.61 ± 2.69	1.797	0.17^a^
Illness duration (months)	6.23 ± 4.63	6.94 ± 3.98		0.544	0.59^c^
HRSD-17 scores	22.69 ± 3.41	20.18 ± 2.67	0.89 ± 0.88	585.979	GI-MDD > nGI-MDD > HC
Anxiety/somatization	7.31 ± 1.92	6.41 ± 1.66	0.39 ± 0.57	174.531	GI-MDD > nGI-MDD > HC
Weight loss	0.80 ± 0.83	0.06 ± 0.24	0	18.741	GI-MDD > nGI-MDD, HC
Cognitive disturbance	3.71 ± 1.78	3.41 ± 1.50	0	64.213	GI-MDD, nGI-MDD > HC
Retardation	6.40 ± 1.42	6.76 ± 1.56	0.18 ± 0.39	253.030	GI-MDD, nGI-MDD > HC
Sleep disturbance	4.46 ± 1.42	3.53 ± 1.28	0.32 ± 0.55	103.570	GI-MDD > nGI-MDD > HC

*GI-MDD, major depressive disorder with gastrointestinal symptoms; HCs, healthy controls; HRSD-17 scores, 17-item Hamilton Rating Scale for Depression; nGI-MDD, major depressive disorder without gastrointestinal symptoms.*

*^a^The p-value was obtained by analyses of variance.*

*^b^The p-value was obtained by a chi-square test.*

*^c^The p-value was obtained by two-sample t-tests.*

### Group Differences

**FIGURE 1:**
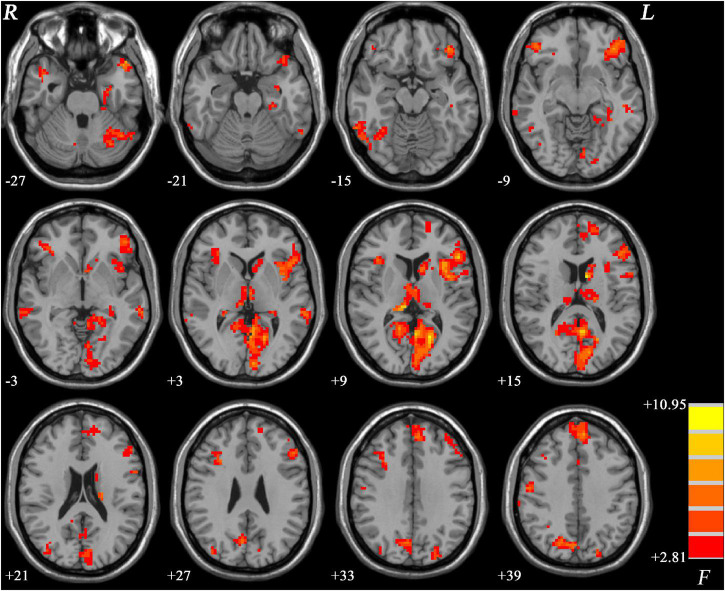
Brain regions showing significantly different PAS scores across three groups. The color bar indicates F values based on ANCOVA. The results were FDR (false discovery rate) corrected at *p* < 0.05. PAS, parameter of asymmetry; ANCOVA, analysis of covariance.

ANCOVA results revealed significant PAS differences in predominantly left hemisphere. Basically, the regions with PAS alterations mainly located in the default mode network (DMN) and left frontoparietal network, including the left parahippocampal gyrus, left caudate, bilateral middle temporal gyrus (MTG), and dorsolateral prefrontal cortex. Widespread regions of visual network such as the fusiform and some parts of the cerebellum also exhibited significant differences (Figure 1).

**FIGURE 2:**
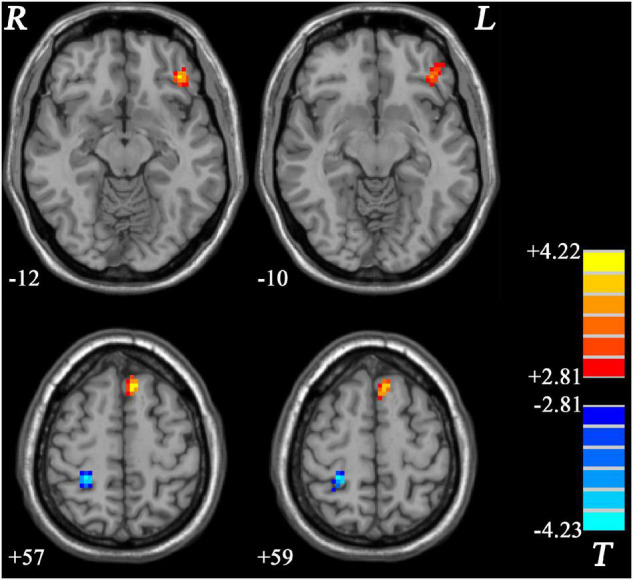
Regions with significantly different PAS scores in comparison between GI-MDD and nGI-MDD patients without GI symptoms. The color bar indicates the *T*-values from *post hoc t*-tests. GI-MDD, major depressive disorder with gastrointestinal symptoms; nGI-MDD, major depressive disorder without gastrointestinal symptoms; PAS, parameter of asymmetry.

GI-MDD patients, relative to nGI-MDD patients, exhibited increased PAS scores in the left inferior frontal gyrus (IFG) and left superior medial prefrontal cortex (MPFC). Moreover, a reduction in PAS was observed in GI-MDD patients in the right postcentral gyrus of the somatomotor network (Table 2 and Figure 2).

**TABLE 2:** Significant PAS differences across groups.

Cluster location	Peak (MNI)			Number of voxels	*T*-value
	*x*	*y*	*z*		
GI-MDD vs. nGI-MDD					
Left IFG	−39	36	−12	61	4.0821
Left superior MPFC	−6	24	57	152	4.2209
Right postcentral gyrus	27	−39	57	67	−4.2251
GI-MDD vs. HCs					
Bilateral PCC/precuneus	−6	−57	15	102	4.0477
Right cuneus	9	−75	42	70	3.9449
Left insula	−33	15	6	76	−4.0040
Bilateral thalamus	9	−30	9	96	−4.2195
Left cerebellum Crus1	−39	−57	−30	42	−3.9492
nGI-MDD vs. HCs					
Left PCC/precuneus	−18	−69	9	361	4.2542
Left IFG/insula	−51	24	9	117	−3.9871
Bilateral superior MPFC	6	36	60	65	−3.6197

*GI-MDD, major depressive disorder with gastrointestinal symptoms; HCs, healthy controls; IFG, inferior frontal gyrus; MNI, Montreal Neurological Institute; MPFC, medial prefrontal cortex; nGI-MDD, major depressive disorder without gastrointestinal symptoms; PCC, posterior cingulate cortex.*

**FIGURE 3:**
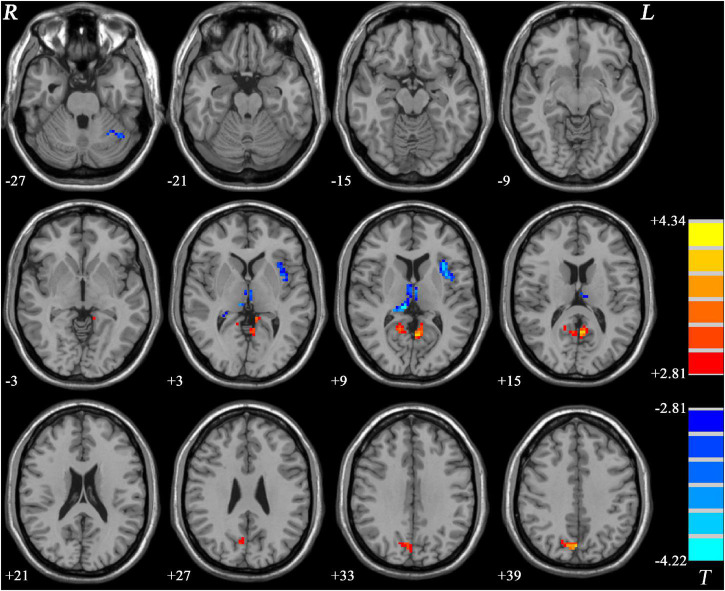
Regions with significantly different PAS scores in GI-MDD patients compared to HCs. The color bar indicates the *T*-values from *post hoc t*-tests. GI-MDD, major depressive disorder with gastrointestinal symptoms; HCs, healthy controls; PAS, parameter of asymmetry.

Comparing to HCs, increased PAS scores were shown in GI-MDD patients in the bilateral posterior cingulate cortex (PCC) and precuneus, which are key components of the DMN. Higher PAS scores were also found in the right cuneus of the frontoparietal network in the GI-MDD group. Left insula, bilateral thalamus, and left cerebellum Crus I showed decreased PAS scores in GI-MDD patients relative to HCs (Table 2 and Figure 3).

**FIGURE 4:**
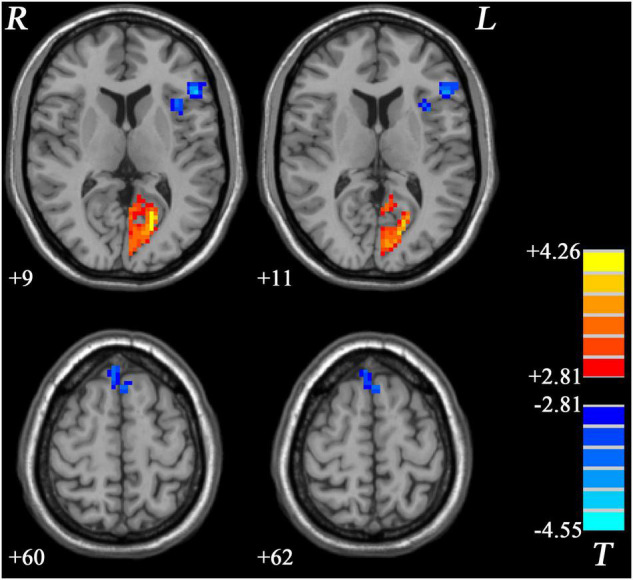
Regions with significantly different PAS scores in nGI-MDD patients compared to HCs. The color bar indicates the *T*-values from *post hoc t*-tests. HCs, healthy controls; nGI-MDD, major depressive disorder without gastrointestinal symptoms; PAS, parameter of asymmetry.

Similar to GI-MDD group, increased PAS scores were found in the left PCC/precuneus in nGI-MDD patients relative to HCs. Decreased PAS scores were displayed in the bilateral superior MPFC, left IFG, and left insula (Table 2 and Figure 4).

### Correlation Analysis

**FIGURE 5:**
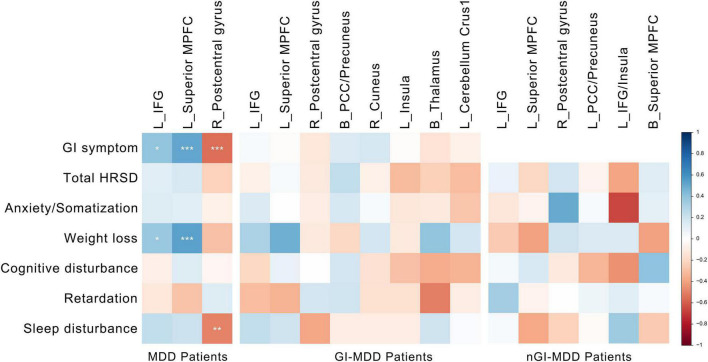
Correlations between altered PAS scores and clinical variables. The color represents the Spearman correlation values. The significance levels are marked with asterisks (*p < 0.05, **p < 0.01, ***p < 0.001). PAS, parameter of asymmetry.

For all MDD patients, their PAS scores of the left IFG and superior MPFC were positively correlated with the severity of GI symptoms and weight loss. Additionally, the severity of GI symptoms and insomnia was negatively correlated to the PAS scores of the right postcentral gyrus (Figure 5). Although potential inverse associations were found between the scores of weight loss and the PAS scores of the right postcentral gyrus, as well as between the severity of retardation symptoms and the PAS scores of the left superior MPFC, their significances did not pass the Benjamini-Hochberg correction.

No correlation was found between clinical variables and PAS scores in GI-MDD or nGI-MDD patients after the Benjamini-Hochberg correction.

### Support Vector Machine Results

**FIGURE 6:**
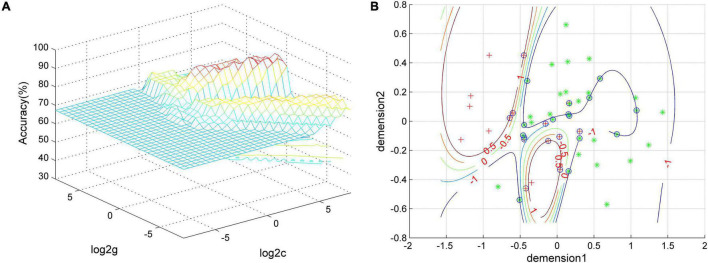
The visualization of SVM classification using the combination of the PAS scores of the left IFG and left superior MPFC. **(A)** SVM parameter result of 3D view and **(B)** classified map of the results. Green asterisks represent the GI-MDD patients, and red crosses represent nGI-MDD patients. GI-MDD, major depressive disorder with gastrointestinal symptoms; IFG, inferior frontal gyrus; MPFC, medial prefrontal cortex; nGI-MDD, major depressive disorder without gastrointestinal symptoms; PAS, parameter of asymmetry; SVM, support vector machine.

Based on the results of group differences between GI-MDD and nGI-MDD, we performed the SVM analysis using the PAS scores of the left IFG, left superior MPFC, and right postcentral gyrus as features. As detailed results shown in Table 3, a combination of the left IFG and left superior MPFC reached a highest accuracy of 92.31%, with a sensitivity of 100% and a specificity of 76.47% (Figure 6). When combining the PAS scores of these three clusters to discriminate the GI-MDD and nGI-MDD patients, the accuracy, sensitivity, and specificity were 90.38, 100, and 70.59%, respectively.

**TABLE 3:** Support vector machine results of discrimination between GI-MDD patients and nGI-MDD patients by using PAS scores.

Brain region(s)	Accuracy	Sensitivity	Specificity
Left IFG	78.84%(41/52)	100%(35/35)	35.29%(6/17)
Left superior MPFC	76.92%(40/52)	85.71%(30/35)	58.82%(10/17)
Right postcentral gyrus	78.85%(41/52)	91.43%(32/35)	52.94%(9/17)
Left IFG + left superior MPFC	92.31%(48/52)	100%(35/35)	76.47%(13/17)
Left IFG + right postcentral gyrus	82.69%(43/52)	97.14%(34/35)	52.94%(9/17)
Left superior MPFC + right postcentral gyrus	84.62%(44/52)	94.29%(33/35)	64.71%(11/17)
Left IFG + left superior MPFC + right postcentral gyrus	90.38%(47/52)	100%(35/35)	70.59%(12/17)

*GI-MDD, major depressive disorder with gastrointestinal symptoms; IFG, inferior frontal gyrus; MPFC, medial prefrontal cortex; nGI-MDD, major depressive disorder without gastrointestinal symptoms; PAS, parameter of asymmetry; SVM, support vector machine.*

## Discussion

This study investigated the effect of GI symptoms on functional asymmetry in first-episode, treatment-naive MDD patients. In comparison across three groups, we found that the regions with significant differences were dominantly distributed in the left hemisphere and involved multiple brain networks including the DMN, visual network, frontoparietal network, and cerebellum. Increased PAS scores in the left IFG and left superior MPFC and decreased PAS scores in the right postcentral gyrus were found in comparison between GI-MDD and nGI-MDD patients. The PAS scores of the left IFG and left superior MPFC were correlated with the severity of GI problems and, more importantly, they could better meet the requirment of discrimination between GI-MDD and nGI-MDD patients. In addition, increased PAS scores in the PCC/precuneus were found in both GI-MDD and nGI-MDD patients.

The nGI-MDD patients showed decreased PAS scores in the left IFG and left superior MPFCs compared to GI-MDD patients and HCs, which indicated both MDD and GI symptoms had potential influence on the alterations of function asymmetry of these regions. Compared to HCs, the nGI-MDD group had lower PAS scores in the left IFG and the bilateral superior MPFC, which indicated that these brain regions had stronger intrahemispheric FC and/or weaker interhemispheric FC possibly related to depression. However, the existence of GI symptoms changed the functional asymmetry of the left IFG and left superior MPFC to the other direction. These results indicated that either increased lateralization or increased interhemispheric connectivity might account for disrupted functions.

Lateralization as a feature occurring in various vertebrate and even invertebrate species was suggested to offer considerable advantages for individuals. For instance, brain functional asymmetry may increase neural capacity by reducing functional redundancy and conflicts between hemispheres (Vallortigara, 2006). In addition, the dominance of one hemisphere can leave the other hemisphere free to have an advantage of other functions, thereby helping to perform parallel processing of functionally separate tasks (Gerrits et al., 2020). Atypical asymmetry was reported to have an association with poor cognitive function (Gerrits et al., 2020) and a variety of neuropsychiatric disorders, such as schizophrenia (Sun et al., 2017), autism spectrum disorder (Carper et al., 2016), and attention deficit/hyperactivity disorder (Ahmadi et al., 2021).

Although the lateralization is more advantageous to process information efficiently, it did not mean that a reduced PAS score is definitely better. The connectivity between the two hemispheres is of great importance. The two hemispheres are connected by commissural system, by which neural information is exchanged between the left and the right half of brain. The commissures were necessary for the construction of functional asymmetry. It was proposed that one hemisphere could send excitatory or inhibitory neural information to the other hemisphere to reduce or enhance functional asymmetry through the commissural system (Bloom and Hynd, 2005). The interhemispheric transfer of multiple types of information, such as perceptual and motor information, would be severely impaired if the corpus callosum was fully severed (Gazzaniga, 2005). Likewise, emotional processing is under the influence of the commissural system. Subjects responded faster and more accurately to the faces with complex social emotions presented in the bilateral visual fields than in unilateral conditions, suggesting the advantage of interhemispheric cooperation in emotional processing (Tamietto et al., 2007). Disrupted interhemispheric connectivity was observed in functional anomalies and multiple psychiatric disorders. The schizophrenic and depressed patients showed altered transcallosal connectivity measured by interhemispheric signal propagation (Hui et al., 2021).

Some researchers proposed that one hemisphere would recruit the other hemisphere to increase processing power when its capability was not adequate for a given task (Banich and Belger, 1990). Therefore, increased interhemispheric connectivity or reduced functional asymmetry might be the manifestation of interhemispheric recruitment for compensation of disturbed functions. It was found in older individuals with functional deficiency of dominant hemisphere that the hemispheric asymmetry reduced (Cabeza, 2002). A study in mice indicated that interhemispheric interaction might be involved in remodeling of cortical plasticity if unilateral cortical sensory input was partially deprived (Jablonka et al., 2021). Another animal study in rats found that the interhemispheric FC was significantly reduced after stroke alongside serious deficits of sensorimotor function and subsequently restored during the recovery period of sensorimotor function (van Meer et al., 2010). A human study reported similar results that interhemispheric interaction played a crucial role in rehabilitation (Chen et al., 2021). When subjects needed to respond to the faces with complex social emotion presented in the left or right visual field, the finding of no difference between unilateral presentations might support the hypothesis of interhemispheric recruitment, because the perception of basic emotion is right-lateralized (Tamietto et al., 2006, 2007). Therefore, increased interhemispheric might be a kind of complementary mechanism to counteract the functional deficiency. Given the perspectives of functional segregation and region-specific process in emotion, the alterations of functional asymmetry in specific regions might give us more information to understand the significance of altered functional asymmetry.

The left IFG and bilateral superior MPFC showed decreased PAS scores in nGI-MDD patients relative to HCs, that is, stronger intrahemispheric FC and/or weaker interhemispheric FC. IFG, a component of the semantic system, also involved in perceiving emotional expressions, is regarded as a region of integrating semantic content and emotional signals in communication. Several neurocognitive studies regarded IFG as a key node in process of affective voices (Schirmer and Kotz, 2006; Belyk et al., 2017). A meta-analysis study of IFG revealed that the perception of semantics was strongly left-lateralized, but emotional perceptions did not show lateralization (Belyk et al., 2017). Therefore, the exacerbated left lateralization of IFG, represented by decreased PAS scores in the left IFG might indicate a dysfunctional emotional perception when perceiving semantics with emotion. The superior MPFC, as a crucial component of the DMN, with close connection to both emotional modulation and interoception, was found to be involved in MDD (Zhou et al., 2010). A previous study investigating interhemispheric connectivity between the homotopic voxels of the two hemisphere using voxel-mirrored homotopic connectivity (VMHC) reported reduced VMHC of MPFC in MDD patients (Guo et al., 2013). The lower VMHC was correlated with poorer performance in Wisconsin Card Sorting Test (Guo et al., 2013). Decreased PAS scores of the superior MPFC in both hemispheres probably was attributed to the reduced interhemispheric connectivity.

However, the occurrences of GI discomfort led to increased PAS scores of the left IFG and left superior MPFC, along with decreased PAS score in the right postcentral gyrus. These aberrant functional asymmetries were not only correlated with the severity of GI dysfunction, but also could draw a clear distinction between GI-MDD and nGI-MDD. The MPFC could be activated by stress and regulated stress-related corticosterone secretion and autonomic function. Additionally, portions of cortical neurons in control of parasympathetic output of stomach are located in MPFC (Levinthal and Strick, 2020). A review concerning stress-induced gastric mucosal lesion has reported the role of MPFC in regulating gastric dysfunction (Zhao et al., 2020). An early study in rats found that bilateral or right lesions, but not the left lesion, of MPFC could reduce plasma corticosterone level and impair gastric stress pathology in the development of gastric ulcer. The lesion of left MPFC could result in stronger emotional and autonomic reactivity in restraint stress, which suggested that the right MPFC facilitated these physiological stress responses and the left MPFC regulated these responses (Sullivan and Gratton, 1999). Therefore, increased PAS scores in the left MPFC might suggest a recruitment to regulate the stress-induced gastric pathology.

The finding of increased PAS scores in the left IFG in comparison between GI-MDD and nGI-MDD patients has intrigued us. A previous study reported that ingestion could increase the cerebral blood flow in the bilateral IFG, implying that IFG might play a role in satiety (Camps et al., 2018). The right IFG was associated with peptide YY, a gut-derived hormone in regulating multiple GI function, including GI motility, secretion, and absorption (Weise et al., 2012; El-Salhy et al., 2020). Moreover, nausea induced by visual stimulation could activate the activity in the right IFG, but activity of the left IFG predominated when using transcutaneous electrical acupuncture to treat nausea (Sarosiek et al., 2017). This issue indicated that functional asymmetry of IFG might be involved in regulation of GI function, and increased PAS scores of the left IFG in our study might be on account of interhemispheric recruitment to regulate the GI dysfunction.

The postcentral gyrus showed significant differences in comparison between GI-MDD and nGI-MDD patients. The postcentral gyrus is a key component of sensorimotor network and contributes to the process of visceral stimulation. The cortical thickness of primary somatosensory cortex was associated with pain intensity in female irritable bowel syndrome (IBS) patients (Grinsvall et al., 2021). Previous studies have reported aberrant activity and function of sensorimotor network in functional GI disorders (Mayer et al., 2009; Duan et al., 2021; Liu G. et al., 2021). Moreover, we observed that altered PAS scores in the right postcentral gyrus had inverse correlation to sleep disturbance in MDD, implying that FC of postcentral gyrus corresponded with sleep quality, which was consistent with previous studies (Huang et al., 2012; Klumpp et al., 2018).

In both GI-MDD and nGI-MDD groups, we found increased PAS scores in the left PCC/precuneus. A previous study of our group using a large sample dataset from the REST-meta-MDD project, the largest MDD resting-state fMRI database, also reported increased PAS scores in the left PCC and precuneus (Ding et al., 2021). These results suggested stronger interhemispheric FC and/or weaker intrahemispheric FC in the left PCC and precuneus in MDD patients. Most studies using VMHC found decreased homotopic connectivity in the PCC and precuneus in MDD patients (Guo et al., 2013; Fan et al., 2018; Shan et al., 2021), though some increased VMHC was reported (Zhao et al., 2021). The inconsistency between these findings and our results might result from different analysis method. The focuses of VMHC and PAS were different. Specifically, the former shows an interest in abnormal interhemispheric FC and only limits to homotopic voxels, whereas the latter expresses interest in the change of functional lateralization. Therefore, it is not surprising that these findings pointed to conflicting results. Both PCC and precuneus are key regions of DMN and were implicated in the introspective processes (Broyd et al., 2009). The altered functional asymmetry in PCC and precuneus suggested less lateralization of these regions in depression.

Moreover, we found decreased PAS scores in the bilateral thalamus, left insula, and cerebellum Crus I and increased PAS scores in the right cuneus in GI-MDD patients compared to HCs, which suggested altered connectivity involved in depression. But these abnormalities of functional asymmetry were only presented in comparison between GI-MDD and HCs, not between nGI-MDD and HCs, suggesting that the occurrence of GI symptoms had influence on functional asymmetry to some extent. Many studies have found that these regions have connections with functional GI disorders (Mayer et al., 2009; Tillisch et al., 2011). The insula was considered as an important region in functional GI disorders, as its role in integrating multimodal sensory information and processing multiple types of information related to visceral sensory, emotional pain, etc. The right insula was considered to have high association with visceral afferent; thus, left-lateralized insula might have adverse effect on the experience of visceral feelings (Mayer et al., 2009). Given that no significant correlation was found between these changes and clinical variables, these abnormalities of functional asymmetry need to be taken with more caution.

Some limitations should be noted. Firstly, we did not categorize the GI-MDD patients based on the types of their GI symptoms in consideration of the small sample size. It would be better to include more nGI-MDD patients since the sample size of GI-MDD group was twice the size of nGI-MDD group. Secondly, this is a cross-sectional study. Although we thought the increased interhemispheric connectivity might be a kind of compensation to counteract the disrupted function, further longitudinal research is required to elucidate that these alterations of functional asymmetry are the cause or the consequence of functional deficiency. Thirdly, as some previous studies, we only used the GI item in the HRSD-17 to evaluate the severity of GI symptoms (Liu et al., 2020; Liu P. et al., 2021). But a more specific evaluation of GI symptoms would be an ideal approach.

## Conclusion

In summary, this study revealed the influence of concomitant GI symptoms on functional asymmetry in MDD patients. Increased PAS scores of the left IFG and superior MPFC might represent unbalanced regulation of brain over GI function and had the potential to be regarded as distinctive features in patients with MDD concomitant GI discomfort.

## Data Availability Statement

The data presented in this study are available upon request to the corresponding author WG.

## Ethics Statement

The studies involving human participants were reviewed and approved by Medical Research Ethics Committee of the Second Xiangya Hospital of Central South University. The patients/participants provided their written informed consent to participate in this study.

## Author Contributions

All authors contributed to and approved the final manuscript. XF wrote the manuscript and contributed to data analysis. YD contributed to the manuscript writing. HL and WG conducted the study. JC, FL, and JZ contributed to data management and analysis. WG designed the study and analyzed the imaging data.

## Conflict of Interest

The authors declare that the research was conducted in the absence of any commercial or financial relationships that could be construed as a potential conflict of interest.

## Publisher’s Note

All claims expressed in this article are solely those of the authors and do not necessarily represent those of their affiliated organizations, or those of the publisher, the editors and the reviewers. Any product that may be evaluated in this article, or claim that may be made by its manufacturer, is not guaranteed or endorsed by the publisher.
